# The Thermal Influence of Oral Rehabilitation on the Cranio-Cervico-Mandibular Complex: A Thermographic Analysis

**DOI:** 10.3390/ijerph181910441

**Published:** 2021-10-04

**Authors:** André Moreira, Ricardo Batista, Susana Oliveira, Joaquim Mendes, Margarida Sampaio-Fernandes, Maria Helena Figueiral

**Affiliations:** 1 Department of Prosthodontics, Faculty of Dental Medicine, University of Porto, 4200-393 Porto, Portugal; ricardomadureirabatista@gmail.com (R.B.); soliveira@fmd.up.pt (S.O.); margaridasampaiofernandes@gmail.com (M.S.-F.); 2 INEGI, Department of Mechanics, Faculty of Engineering, University of Porto, 4200-465 Porto, Portugal; jgabriel@fe.up.pt; 3 INEGI, Department of Prosthodontics, Faculty of Dental Medicine, University of Porto, 4200-393 Porto, Portugal; hfigueiral@gmail.com

**Keywords:** cranio-cervico-mandibular complex, infrared thermography, oral prostheses, oral rehabilitation, prosthodontic

## Abstract

Purpose: Assess the thermal effect of prosthodontic treatment on the cranio-cervico-mandibular complex using infrared thermography. Methods: The treatment group was composed of adults of both sexes who underwent a prosthodontic treatment in which at least posterior occlusal contacts were added and/or the vertical dimension of occlusion was reestablished. The control group (CG) was constituted of adult subjects of both sexes, with no more than a single missing posterior tooth, excluding third molars. Thermograms were taken of the treatment group with a Flir i7 IR camera both before oral rehabilitation (TGB; *n* = 33) and two months after treatment was concluded (TGA; *n* = 19). CG (*n* = 33) had only one occasion for data acquisition. Results: Statistically significant differences were found when the thermal difference (ΔT) and the health status of the orbicularis oris muscle were compared between the TGB and the TGA groups (*p* = 0.020 and *p* = 0.003, respectively). By comparing the health status of the masseter muscle between the CG and TGB, statistically significant differences were also observed (*p* = 0.030). Conclusion: A prosthodontic treatment appears to have a minimum or null effect on the ΔT and/or on the health status of the TMJ and the temporal muscle. In contrast, orbicularis oris muscles exhibited significant thermal variations.

## 1. Introduction

The cranio-cervico-mandibular complex (CCMC) is composed of multiple structures, as diverse as bones, ligaments, and muscles. In humans, the CCMC includes four main groups of muscles: muscles responsible for the mandibular movement, muscles of the tongue, facial muscles, and neck muscles [1]. In addition, there are two temporomandibular joints (TMJ)—the right and the left—where an intra-articular disc articulates with the condylar process of the mandible below, and the mandibular fossa of the temporal bone above. The TMJ is protected by a vast number of ligaments, which limits the movement of the mandible [2]. Teeth, located in the maxillary and mandibular alveolar processes, are also involved in the CCMC. Contraction of the jaw elevator muscles allows intercuspation, thus creating a third supporting point between the cranium and the mandible. Besides their importance for the mandibular cinematic (incisal and lateral guidance, Spee and Wilson curves, and vertical dimension of occlusion (VDO)), teeth are also crucial for mastication, phonetics, and aesthetics [3,4]. It therefore comes as no surprise that tooth loss or misalignment may result in occlusion-related problems. In fact, a lack of tooth contacts and an incorrect relationship between the cranium and the mandible may have a negative impact on other structures of the CCMC [5,6].

Temporomandibular disorders (TMD) are a sub-group of musculoskeletal disorders (MSD) that are characterized by signs and symptoms affecting the TMJ, the masticatory muscles, and other structures of the CCMC. TMD etiology is usually multifactorial, and conditions such as attrition, erosion, edentulism, misfit oral rehabilitations, and incorrect VDO may act as predisposing factors [7,8,9]. It should be highlighted, however, that thanks to the broad adaptability of the human body, masticatory dysfunction may be present without clinical signs and symptoms of TMD [3]. Masticatory force usually increases after fixed implant-supported oral rehabilitations [10,11]. Besides, it has been described that an improvement in masticatory force may be correlated with enhanced muscular activity [12,13,14]. A number of studies show that oral rehabilitation in edentulous patients with implant overdentures or fixed implant dentures leads to increased electromyographic activity of masticatory muscles, namely the masseter and temporal muscles [15,16,17,18]. In addition, other authors suggest that the electromyographic activity of oral rehabilitated patients is similar to that of dentate subjects [19]. In contrast, Goiato et al. refer to a decrease in the electromyographic activity of the temporal and masseter muscles after 5 months of using new complete dentures with restored VDO [20].

Infrared thermography (IRT) was introduced into the medical field in the 1960s to evaluate and quantify body temperature. Since then, it has been used to record skin temperature, which is related to the microvascular dynamics of the anatomical structures lying underneath [21,22,23]. Thermography is advantageous because there is an absence of contact during examination, infrared is a form of non-ionizing radiation, and it is fast, duplicatable, and low cost. There are two main forms of thermographic examination in medicine: static (a simple image acquisition) and dynamic (acquisition of multiple images after a stimulus). IRT has proven useful in the examination of neuropathies, breast cancer, peripheral vascular disorders, myofascial syndromes, fever screening, and dermatology. It has been documented that IRT constitutes a valid complementary diagnostic tool for TMD and orofacial pain [22,24]. A reliable method for assessing the health status of a region of interest (ROI) is using the thermal difference (ΔT) of right vs. left side anatomical structures (ΔT = |Left ROI–Right ROI|) [25,26]. The ΔT cutoff point that is defined to differentiate healthy and abnormal structures depends on the ROI analyzed. Concerning orofacial structures, previous findings have established that a value between 0.4 °C and 0.5 °C is the most accurate cutoff point (ΔT ≥ 0.4 °C—abnormal; ΔT < 0.4 °C—healthy/normal) [27].

Based on the evidence described above, this study aims to assess the thermal effect of a prosthodontic treatment on the CCMC components (anterior part of the temporal, masseter, and orbicularis oris (Oo) muscles, and TMJ) of edentulous subjects (complete edentulous and Kennedy class I and II partially edentulous patients). Our null hypothesis in this research is that prosthodontic rehabilitation has no thermal influence on the orofacial structures of interest.

## 2. Materials and Methods

### 2.1. Ethics

The present study was conducted in accordance with the World Medical Association Declaration of Helsinki and was approved by the ethics committee of the Faculty of Dental Medicine of the University of Porto (FMDUP), no. 000087. Written consent, explaining the objectives and methodology of the study, was obtained from all participants involved. Data were anonymized and participants could withdraw at any moment.

### 2.2. Sample

**Figure 1:**
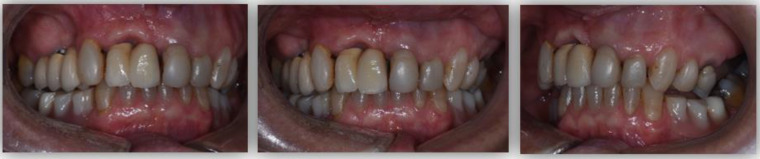
Intra-oral photographs of a participant from the treatment group before the oral rehabilitation (TGB): (**a**) right view; (**b**) frontal view; (**c**) left view.

**Figure 2:**
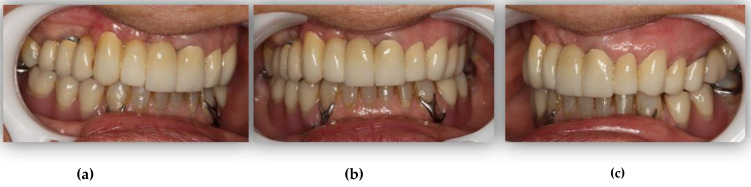
Intra-oral photographs of the patient depicted in Figure 1, after oral rehabilitation (TGA): (**a**) right view; (**b**) frontal view; (**c**) left view.

GPower 3.1. software was used to determine the sample size appropriate for the statistical tests, with a significance level (α) of 5%. The software recommended a sample size of 47 participants per group; however, for the present pilot study, both the control and study groups were smaller (*n* = 33) due to inherent limitations. The treatment group was composed of 33 patients who underwent oral rehabilitation at the clinic of FMDUP between 2017 and 2020 (TGB; *n* = 33). The included prosthodontic treatments comprise conventional removable dentures and tooth- or implant-supported fixed dentures that added at least two posterior occlusal contacts and/or reestablished the VDO (complete edentulous and Kennedy class I and II partially edentulous patients; Figure 1 and Figure 2). Subjects who met any of the following criteria were excluded: age < 18 years, the presence of TMD, ongoing endodontic treatment, recent oral surgery (<6 weeks), and the presence of degenerative diseases. For TMD screening, we used clinical examination complemented by the 3Q/TMD. This questionnaire has been indicated for trials due to its simplicity and high sensitivity (0.81–0.96) [28,29].

The number of participants assessed after prosthodontic treatment decreased to 19 individuals (TGA; *n* = 19) for the following reasons: (a) 5 participants did not conclude the treatment; (b) 3 participants were not able to return to FMDUP; (c) 2 participants were uncontactable; and (d) 4 participants needed multiple adjustments of the new removable dentures, the conclusion of which exceeded the data acquisition period.

**Figure 3:**
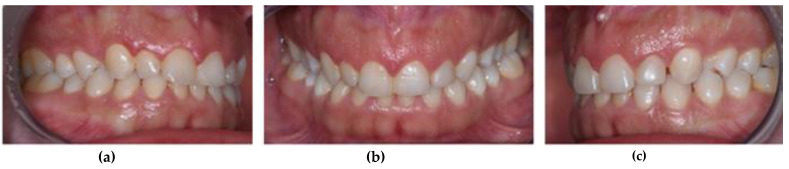
Intra-oral photographs of a participant from the control group (CG): (**a**) right view; (**b**) frontal view; (**c**) left view.

The control group (CG) was composed of 33 participants of both sexes, with no more than a single missing posterior tooth, excluding third molars (Figure 3). The exclusion criteria for the control group were the same as those used for the treatment group.

### 2.3. Thermographic Procedure

**Figure 4:**
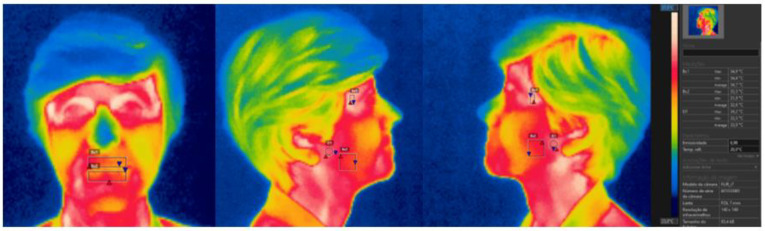
Frontal, right lateral, and left lateral views of the thermograms analyzed with the Flir Tools^®^ software.

The infrared images were obtained with a Flir i7 IR Camera 140 × 140 Resolution/9 Hz from Flir^®^ Systems Inc. (Wilsonville, Oregon, USA.), using an emissivity value of 0.98 (the value recommended for the skin). Image processing was performed with the Flir Tools, Flir^®^ Systems Inc. (Wilsonville, Oregon, USA.) software, and involved the selection of a representative area (polygon) of the ROI (Figure 4). Prior to the thermographic recordings, participants were instructed to abstain from heavy meals, alcohol, coffee, tea, medications, and smoking for 2 h before image acquisition, in order to prevent metabolic phenomena that could affect the skin’s temperature. The use of oils, creams, or makeup on the regions to be imaged was also not allowed, and males were instructed to shave the day before the exam [30,31,32]. The room was acclimatized for 15 min prior to the examination. To prevent measurement errors, the IRT camera was positioned at a 90° angle, 1 m away from the anatomical ROI of participants, who maintained a static posture during the image acquisition. The treatment group was examined both before the prosthodontic treatment (TGB) and two months after its conclusion (TGA), whereas the control group (CG) was only assessed once. To minimize camera-related errors, the head and neck region of each subject was recorded twice for each of the frontal, left, and right lateral views. The average value of both thermograms (expressed as degrees Celsius) was used for data analysis. A total of 510 thermograms were obtained, 6 per participant.

### 2.4. Data Analysis

For each group (CG, TGB and TGA), a dataset containing the average temperature value, the right vs. left side thermal difference (ΔT), and the health status of each ROI (Oo, masseter, temporal, and TMJ) was created. Statistical analysis was performed using SPSS v24 (IBM^®^, Armonk, NY, USA) software. The normal distribution of variables was assessed using the Kolmogorov–Smirnov and Shapiro–Wilk tests. Since all variables followed a non-normal distribution (*p* < 0.05), non-parametric tests were used. Accordingly, to compare the ΔT of the ROIs of TGB vs. TGA, the Wilcoxon test was used (paired samples), while the ΔT of the ROIs of CG vs. TGB and of CG vs. TGA were compared using the Mann–Whitney U test (independent samples). To compare the health status of each ROI between groups, a chi-square test was used. An alpha value of 0.05 was considered.

## 3. Results

### 3.1. Thermographic Characterization of the CCMC Structures

The mean values of the average temperature (Tm) obtained for each ROI (two thermograms per view) are shown in Table 1. Since potential biases could affect the direct comparison of the Tm between groups (see Discussion below), the absolute values of Tm were not statistically analyzed. In the three groups, however, the temporal muscles were the structures with the highest mean temperature, followed by the TMJ.

### 3.2. Comparative Analysis of the Thermal Difference (ΔT) of the CCMC Structures

The thermographic characterization of each ROI was complemented by assessing the corresponding ΔT value, which compares the Tm measured in homolog contralateral structures, or in upper vs. lower structures, as is the case with the Oo muscle.

**Figure 5:**
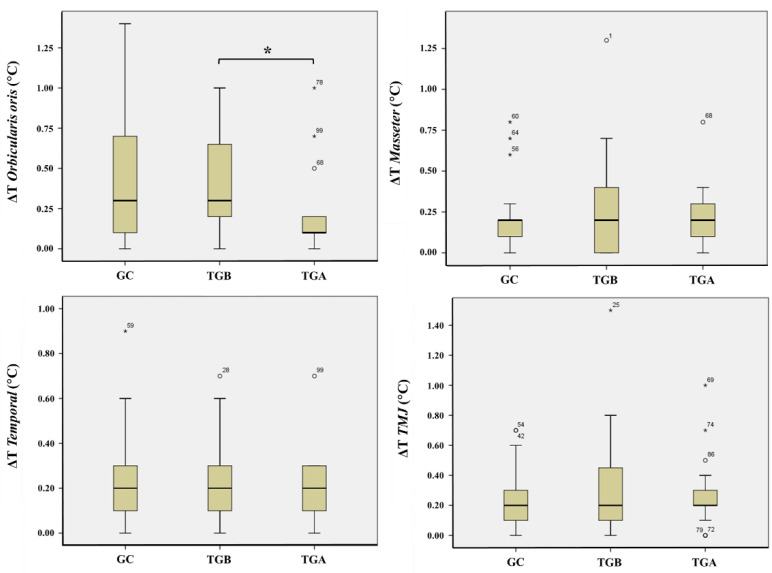
Box-and-whisker plots of the thermal difference (ΔT) for each ROI of the control group (CG) and treatment group, before and after oral rehabilitation (TGB and TGA, respectively). The asterisk (*) mark the statistical difference found for the Oo when comparing TGB with TGA.

As Figure 5 depicts, a statistically significant difference was only found for the Oo muscle, with the asymmetry level decreasing after oral rehabilitation of patients (TGB vs. TGA, *p* < 0.05). For the remaining intra- and inter-group comparisons of ΔT values, no statistically significant differences were observed. Detailed results of the statistical analyses performed are summarized in Table 2.

### 3.3. Comparative Analysis of the Health Status of the CCMC Structures

Assuming ΔT = 0.4 °C as the cutoff point (27), the frequency of healthy and abnormal structures was evaluated (Table 3). Overall, the number of abnormal ROIs appear to decrease after oral rehabilitation of patients (TGA) in comparison with the initial condition (TGB), with the TMJ being the exception.

For a quantitative analysis of these results, a chi-square test was used (Table 4). Comparing the CG and TGB groups, only the masseter muscle showed statistically significant differences with regard to health status (*p* = 0.030), with a higher number of abnormal structures occurring in edentulous patients prior to the prosthodontic rehabilitation. Such difference was not observed when the CG was compared to the TGA, and this pattern was also found in the remaining orofacial regions studied.

Finally, the TGB vs. TGA intra-group comparison revealed statistical differences for the health status of the Oo muscle, with the frequency of abnormal structures significantly decreasing after oral rehabilitation (*p* = 0.003). The other ROIs (masseter, temporal and TMJ) showed no statistical differences between the TGB and TGA.

## 4. Discussion

The null hypothesis that prosthodontic rehabilitation has no thermal influence on orofacial structures was partially rejected, as for the masseter and Oo muscles statistically significant differences were observed, while for the temporal muscle and for the TMJ, no temperature-related changes were found.

IRT has been recognized as a valuable method for skin temperature assessment in the medical and occupational fields, being routinely used as a complementary diagnostic tool in oncology (skin and breast, among others), MSD, lower back pain, vascular disorders, rheumatic disorders, and in sports medicine [32]. This technique is particularly useful in clinical conditions in which pain and expected thermal cutaneous alterations coexist. In fact, skin temperature can be assessed and presented in absolute values, normal values, or as thermal difference. IRT may detect vascular and/or nervous alterations occurring in underlying structures [24]. Although there are multiple studies evaluating IRT as a diagnostic tool for TMD, to the best of our knowledge, the present study is the first of its kind to use thermographic analysis in the context of oral rehabilitation.

In the current study, participants with signs or symptoms of TMD were excluded. As the temperature of the ROI could be affected by TMD-related phenomena, this criterion was an attempt to preclude potential confounding variables and misinterpretation of the results. Infrared imaging reveals the skin temperature landscape, which enables the comparison of bilateral homolog structures in terms of thermal symmetry. According to the literature, thermal differences (ΔT) greater than or equal to 0.4 °C may be considered pathological in the head and neck regions. Despite IRT imaging having been conducted under standardized conditions and protocols, the absolute values of Tm can be affected by multiple endogenous and exogenous variables beyond our control. In line with other studies, the statistical data analysis presented herein relied on ΔT of homolog structures, rather than on the absolute Tm of the ROI.

Although not reflected in the ΔT values, the number of abnormal masseter muscles was significantly increased in the TGB when compared with the CG; however, such difference was not observed when patients were examined after the prosthodontic treatment. These opposite findings make it unclear whether an oral rehabilitation procedure improves the health status of the masseter muscle. No information regarding electromyographic activity can be inferred from the present study.

Concerning the Oo muscle, our data revealed a statistically significant decrease in thermal asymmetry after oral rehabilitation of patients, a result also paralleled by a significant reduction in the number of abnormal structures (TGB vs. TGA). The impact of prosthodontic treatment on this muscle has also been evaluated by electromyography and may provide important clues for our observations. In fact, using a group of 15 participants, Caxias et al. reported that new, complete dentures decreased the electromyographic activity of the Oo muscle while in resting position, with the lower part being more active than the upper [33]. Similar results were obtained by Santos et al., using electromyographic tests under two clinical conditions, both before and after the placement of complete dentures. Their results showed higher electromyographic readings in the lower fascicle of the Oo muscle [34]. It should be highlighted that, in our experimental conditions, thermograms were collected in a resting position, with no chewing exercises preceding the examinations. However, minimal electromyographic activity remains positive even in a resting position, which maintains a basal muscle tone.

Myofunctional therapy and interceptive orthodontics can be re-educational and have the ability to balance muscular forces, thus improving the activity of the stomatognathic system [35]. It is therefore conceivable that oral rehabilitation procedures may offer identical benefits. Saccucci et al. reported changes in the Oo’s electromyographic activity after an interceptive orthodontic treatment. The work reported that muscle activity was increased in the inferior part of the muscle in resting position and in the superior part during mandible protrusion [36].

As previously mentioned, a decline in masticatory function is expected after tooth loss; thus, occlusion is a pivotal factor influencing the nutritional state of geriatric patients. Besides, there is long-standing recognition that tooth loss is accompanied by a maxillary reduction in the superomedial direction, which impairs the support of soft tissues. The absence of anterior teeth compromises not only facial aesthetics but also the proper lip seal and other labial functions. Hence, it is reasonable that restoring lip support with a well-fitted removable or fixed denture may positively influence muscular activity and consequently the thermal symmetry of the Oo muscle, thus explaining our findings; however, the contribution of the lower part of the Oo to this observation cannot be ruled out.

The thermographic analysis of both the TMJ and the anterior portion of the temporal muscle provided no statistically significant results, suggesting that the thermal pattern of these structures is not particularly affected by edentulism, nor by prosthodontic treatments. As TMD patients were excluded from this study, abnormalities typically related to intracapsular disorders, lupus, and rheumatoid arthritis—in which the temperature is influenced by the progression and severity of the disease—were not found during the IRT image processing.

Although important insights have been provided by this pilot clinical study, some limitations are worth noting. The sample size was smaller than that recommended by the power calculations. The IR camera resolution is a present limitation, since better IR camera resolution would offer even more accurate results. The number of participants assessed after oral rehabilitation (TGA) was lower than that of the initial group (TGB), which compromised the homogeneity of the sample size. To find candidates meeting the inclusion criteria and willing to participate is demanding, involving the investment of time and the collaboration of multiple professionals. Increasing the sample size of all groups should be guaranteed in further studies, since it will enable to create additional sub-groups based on age, gender, or the type of prosthodontic treatment (e.g., removable vs. fixed, complete vs. partial, anterior vs. posterior, among others), thus refining results and conclusions. Since the control group was not assessed a second time, it was not possible to determine the measurement error of the technique. Six- and twelve-month follow-up timepoints would bring more insights to the topic. Additionally, DC-TMD is a more rigorous examination protocol than 3Q-TMD and it would have been mandatory if we had included TMD patients.

## 5. Conclusions

Despite some limitations, the results of the present study suggest that the Oo muscles are structures of the CCMC, susceptible to thermal variations elicited by prosthodontic treatments. Comparatively, the TMJ and temporal muscles showed minimal or null thermal changes upon oral rehabilitation of patients. No clear conclusions were reached regarding the masseter muscle being influenced thermally by the prosthodontic treatment.

Although the electromyographic activity of masticatory muscles before and after oral rehabilitation has been extensively described elsewhere, thermal imaging assessment remains poorly explored in this context. The occlusal balance and the redistribution of masticatory forces provided by the prosthodontic treatment may influence the temperature of some CCMC structures. In line with our findings, the systematic recording of thermographic data during the prosthodontic workflow may constitute an interesting complementary strategy to monitor the quality of oral rehabilitation.

## Figures and Tables

**Table 1:** Mean values of the average temperature (Tm) recorded in the thermograms of the studied ROI for the CG, TGB and TGA groups.

Group ROI	(Mean Values of Tm; Mean ± SD, °C)		
	CG (*n* = 33)	TGB (*n* = 33)	TGA (*n* = 19)
Superior Oo	32.86 ± 2.032	32.93 ± 0.946	32.87 ± 1.164
Inferior Oo	32.97 ± 2.017	33.92 ± 0.961	32.94 ± 1.271
Right Masseter	32.89 ± 1.854	32.79 ± 1.078	32.87 ± 1.218
Left Masseter	32.91 ± 1.862	32.84 ± 0.908	32.96 ± 1.209
Right Temporal	34.45 ± 1.562	34.03 ± 0.554	34.05 ± 0.927
Left Temporal	34.43 ± 1.418	34.00 ± 0.576	34.08 ± 0.938
Right TMJ	33.24 ± 1.863	33.12 ± 0.825	32.96 ± 1.266
Left TMJ	33.27 ± 1.832	33.08 ± 0.729	33.11 ± 1.154

CG—control group; TGB—treatment group before oral rehabilitation; TGA—treatment group after oral rehabilitation; SD—standard deviation.

**Table 2:** Statistical data analysis of the intra- and inter-group comparisons of the ΔT values of each ROI.

Variable Tested	TGB × TGA (Wilcoxon)	CG × TGB (Mann–Whitney U)	CG × TGA (Mann–Whitney U)
ΔT Oo	0.020	0.301	0.085
ΔT Masseter	0.270	0.724	0.233
ΔT Temporal	0.063	0.611	0.975
ΔT TMJ	0.204	0.823	0.173

CG—control group; TGB—treatment group before oral rehabilitation; TGA—treatment group after oral rehabilitation.

**Table 3:** Number of healthy and abnormal ROIs assessed in the CG, TGB, and TGA, considering ΔT = 0.4 °C as the cutoff value.

Variable Tested	Health Status of the ROI		
	CG (Healthy/Abnormal)	TGB (Healthy/Abnormal)	TGA (Healthy/Abnormal)
Oo	20/12	15/18	15/2
Masseter	30/3	23/10	14/5
Temporal	30/3	29/4	19/0
TMJ	27/5	24/9	14/8

CG—control group; TGB—treatment group before oral rehabilitation; TGA—treatment group after oral rehabilitation.

**Table 4:** Chi-square analysis comparing the health status of the ROI (healthy/abnormal) between groups.

Variable Tested	TGB × TGA (Chi-Square Test)	CG × TGB (Chi-Square Test)	CG × TGA (Chi-Square Test)
ΔT Oo	0.003	0.168	0.058
ΔT Masseter	0.760	0.030	0.097
ΔT Temporal	0.114	0.689	0.176
ΔT TMJ	0.940	0.253	0.353

CG—control group; TGB—treatment group before oral rehabilitation; TGA—treatment group after oral rehabilitation.

## Data Availability

Data regarding this study can be shared upon request to the corresponding author.
